# Patients’ Knowledge and Perceptions Towards Digital Technologies in Dentistry: A Cross-Sectional Study

**DOI:** 10.3390/dj13120569

**Published:** 2025-12-02

**Authors:** Aliona Dodi, Alecsandru Ionescu, Mihaela Anca Marin, Marina Imre

**Affiliations:** 1 Discipline of Prosthodontics, Faculty of Dentistry, “Carol Davila” University of Medicine and Pharmacy, 37 Dionisie Lupu Street, District 2, 0200221 Bucharest, Romania; mihaela.marin@umfcd.ro (M.A.M.); marina.imre@umfcd.ro (M.I.); 2 Pedodontics Department, Faculty of Dentistry, “Carol Davila” University of Medicine and Pharmacy, 37 Dionisie Lupu Street, District 2, 0200221 Bucharest, Romania; alecs.ionescu@adental.ro

**Keywords:** digital technologies, dentistry, survey, patient perception, socio-demographic factors, acceptance, modern treatments

## Abstract

**Background/Objectives:** The accelerated digitalisation of dental practice is significantly influencing how patients perceive and accept modern treatments. This study uses a structured questionnaire to evaluate patients’ knowledge of, and attitudes towards, digital technologies in dentistry, adopting an original, patient-centred perspective within routine clinical settings. **Methods:** Non-parametric statistical methods (Mann–Whitney U test, Kruskal–Wallis H test and Spearman correlations) were employed to analyse the responses of 397 participants. To reduce selection bias, a systematic sampling technique was employed, and thorough validation ensured the consistency of the instrument. The questionnaire covered socio-demographic information, prior dental experience and opinions regarding specific digital applications (intraoral scanning (IOS), cone-beam computed tomography (CBCT), CAD-CAM workflows, 3D printing). Knowledge was operationalised as awareness; no keyed objective knowledge test was administered. **Results:** The findings show that patients generally accept digital technologies, with perceptions of costs, prior experience of digital dental procedures and educational level having a significant impact. The duration of the patient–clinician relationship, the patient’s dental health, and the history of orthodontic and prosthetic procedures also impacted the acceptance of digital technologies. Notably, clinical staff members were the main source of information, highlighting the importance of professional–patient communication. **Conclusions:** The results highlight the importance of patient-friendly communication in healthcare and provide a solid basis for the implementation of patient-centred digital dentistry. Future plans should focus on creating specialised educational materials, improving digital literacy, and promoting equal access to cutting-edge technologies in urban and disadvantaged communities.

## 1. Introduction

One of the most significant developments in modern dentistry is the adoption of digital technologies, which have a substantial impact on accuracy, clinical efficiency and the patient experience [1,2]. Technologies such as intraoral scanning, CAD/CAM design, 3D printing and artificial intelligence are increasingly being used in prosthetic rehabilitation and surgery [3,4,5]. Furthermore, general healthcare trends indicate that patients are increasingly receptive to the use of digital tools for monitoring and communication purposes [6].

Beyond technical performance, however, the clinical uptake of these technologies depends on patients’ acceptance and perceived value. In routine care, patients tend to balance perceived benefits—greater comfort and fewer impressions with intraoral scanning, streamlined chairside efficiency with CAD/CAM, improved planning and visualisation with CBCT, and decision support with AI-assisted systems—against concerns such as CBCT radiation, data privacy for AI, and the durability or cost of chairside CAD/CAM solutions [1,3,4,5,6]. Acceptance is also shaped by how information is communicated and by the patient’s ability to understand, appraise and use it; therefore, digital literacy is a relevant determinant alongside clinical considerations [4,7,8,9,10,11]. Across applications, patient acceptance, shaped by comfort with intraoral scanning, informed risk–benefit understanding for CBCT, usability and satisfaction with teledentistry, and transparent clinician oversight when AI is used, has been shown to condition the adoption of digital workflows in practice [6,10,11]. Consequently, finding out how patients feel about digitalisation is an important part of creating modern, patient-centred dentistry.

Recent studies show that an increasing number of people are accepting digital technologies, although this varies according to age, education level, prior medical/dental experiences and sources of information [12,13,14,15]. People may perceive these new technologies as more useful and safer if they can access information about them online and consult with professionals in the field [16,17]. The use of teledentistry and other related tools has also increased due to the impact of the pandemic, changing what patients want and expect regarding accessibility and communication in dental care, potentially modulating attitudes towards digital workflows [18,19].

Despite substantial research on individual digital applications, there is limited observational evidence that integrates patients’ knowledge and prior exposure (intraoral scanning, CBCT, CAD/CAM), digital/e-health literacy and information sources, with basic clinical history (e.g., number of natural teeth; prior orthodontic/prosthetic treatments) within routine practice. This evidence gap reduces the interpretability and generalisability of patient-centred implementation strategies in everyday dental care. In this situation, closing this gap is important so that clinical decisions and communication are aligned with the factors that shape patients’ acceptance and intention to use digital technologies.

The aim of this study is therefore to systematically investigate patients’ attitudes towards the digital technologies used in modern dental treatments (intraoral scanning, CBCT, CAD/CAM) in routine clinical settings, considering both sociodemographic and clinical factors that influence their willingness to accept them. Based on prior reports, we expected basic familiarity with digital dentistry, clinician-led information seeking, and generally favourable attitudes towards clinics employing modern technologies, even at higher cost. The research questions were as follows:

What is the level of knowledge and prior exposure to digital dental technologies among patients, and which sources of information do they report?

What is the profile of attitudes and perceptions towards digital dental technologies in routine care—comfort, perceived safety, accuracy, treatment duration, invasiveness, and cost/benefit trade-offs—including clinic preference for providers using modern technologies?

Are knowledge and attitudes associated with sociodemographic characteristics?

Are knowledge and attitudes associated with clinical history and engagement with dental care?

Is prior exposure to/knowledge of specific applications associated with greater acceptance and intention to use, including preference for clinics using new technologies and willingness despite higher costs?

## 2. Materials and Methods

The study was conducted from 30 September 2024, to 6 February 2025, and it used an observational, descriptive, and cross-sectional design. The study was conducted in accordance with the Declaration of Helsinki and approved by the Scientific Research Ethics Committee and the Scientific Council of the “Carol Davila” University of Medicine and Pharmacy (UMFCD), Bucharest, Romania (Ethics Committee Approval No. 16344, Date 1 July 2025). This study design enables precise measurement of the prevalence and distribution of patients’ attitudes towards digital technologies in dentistry at a given moment in time, looking into the links between sociodemographic factors and attitudes without needing longitudinal follow-up of participants. It is also cost-effective and time-efficient.

Three private dental clinics (two in Bucharest and one in Galati) used a self-administered digital questionnaire to collect data. Participants completed the survey either on a tablet during their visit to the clinic or online using a unique link that was sent to them by the clinic staff after they had provided informed consent. All three participating sites were urban clinics; therefore, generalisability to non-urban (including rural) populations may be limited.

The selection of participants was conducted through the utilisation of systematic sampling: every third patient scheduled on the daily appointment lists at each participating clinic was invited to participate. If an eligible invitee declined or did not meet inclusion criteria, the next eligible scheduled patient was invited, and the fixed interval was then resumed. This approach resulted in a more consistent recruitment process across sites and periods and sought to mitigate the risk of selection bias. However, it did not achieve randomisation of participants, which is acknowledged in the study limitations.

### 2.1. Inclusion and Exclusion Criteria

Patients were included in the study if they were at least 18 years old, attended one of the three participating dental clinics during the study period, were able to understand and complete the digital questionnaire, and gave their informed consent. Patients were excluded from participation if they did not speak Romanian, exhibited severe cognitive impairments that precluded them from completing the questionnaire, or declined to participate.

### 2.2. Data Collection Tool

The questionnaire comprised a number of sections, including enquiries into the subject’s sociodemographic background and prior dental experiences (Q1–Q19), level of knowledge, and attitude towards digital technologies in contemporary dentistry (Q20–Q34). The Likert scale employed in this study comprised five steps, ranging from 1 (strongly disagree) to 5 (strongly agree). The selection of this scale was based on finding the best balance between psychometric sensitivity and the ease with which respondents could engage with the instrument. Research shows that a five-point scale ensures the reliability of responses while lowering the risk of cognitive fatigue and ambiguity [20,21].

In this study, knowledge was operationalized as awareness. Specifically, items captured self-reported exposure, familiarity, and recognition of key digital domains (intraoral scanners, CAD/CAM, 3D printing, guided surgery). The instrument did not include a keyed objective knowledge test. Accordingly, analyses and inferences regarding “knowledge” are restricted to awareness indicators.

We prospectively screened the Likert blocks for undifferentiated response patterns (e.g., identical answers across multiple scaled items). Handling rules are detailed under Statistical analysis.

Two questionnaires showed partial missing data for scaled items (Q20–Q34). These cases were handled as specified under Statistical analysis.

### 2.3. Questionnaire Validation

The instrument was validated using an integrative methodological approach that included content validation, checking for internal consistency, finding response bias, and looking at the correlations between items.

Validating the content

Five experts (two dentists, two academics, and a dentist with experience in digital dental technologies) evaluated for clarity, relevance and conceptual coherence. A Content Validity Index (S-CVI/Ave) of 0.92 was found based on these tests, suggesting very good content validity [22,23].

Internal consistency

We used Cronbach’s Alpha coefficient = 0.926 to test the internal reliability of the scaled items (Q20–Q34). This was performed on a sample of 395 fully filled out questionnaires. Based on the standards set by George & Mallery and DeVellis & Thorpe [24,25], this value exhibits excellent internal consistency.

Data cleaning and finding response bias

Thirty-one questionnaires (7.8%) were found in which all 15 Likert scale items received the same answer from the respondents and forty questionnaires in which respondents gave the same answer (e.g., “5”) to all scaled items Q22–Q34, possibly due to an automated pattern or straightlining. According to methodological advice on undifferentiated responses [26,27,28], these questionnaires could change the mean and variance of the scores and were marked for potential sensitivity analyses. To ensure the statistical validity of the results, these questionnaires were included in the overall descriptive analysis to reflect the full distribution of perceptions but were excluded from the comparative and correlational analyses (Mann–Whitney, Kruskal–Wallis, Spearman), because identical responses do not provide variance and may bias the coefficient estimates. This balanced approach has allowed statistical integrity to be maintained and provides as accurate a representation as possible of genuine patient perceptions.

Validity of the construct

Construct validity was supported by:

- Comparisons between groups showed that there were statistically significant differences by age, previous experience with digital treatments, and marital status.
- Spearman correlations between items Q20 and Q34, with coefficients ranging from r = 0.18 to r = 0.74, showing convergent validity [29,30].

Response coding

The scaled items were transformed numerically in the following ways: 1 = Strongly disagree, 2 = Disagree, 3 = Neutral, 4 = Agree, 5 = Strongly agree. This coding made it possible to use non-parametric statistical tests.

### 2.4. Statistical Analysis

The study used IBM SPSS Statistics, version 25.0 (IBM Corp., Armonk, NY, USA) to analyse the data. Microsoft Excel and Word 2024 (Microsoft Corp., Redmond, WA, USA) were used to illustrate the data. The choice of statistical tests and interpretation of results were performed according to methodological recommendations [30]. Qualitative variables were shown as percentages or frequencies. We used means with standard deviations or medians with interquartile ranges to show quantitative variables. The Shapiro–Wilk test was used to check if the distribution of quantitative variables was normal. Mann–Whitney U and Kruskal–Wallis H tests (with post hoc Dunn-Bonferroni tests) were used to compare independent quantitative variables with non-parametric distributions between groups. The Spearman correlation coefficient (rho) was used to estimate the correlations between quantitative independent variables. For all tests, the level of significance was set at α = 0.05. There were 397 valid questionnaires in total, two of which had missing answers for some scaled items (Q20–Q34). These cases were handled via pairwise deletion (included where responses existed; excluded only when complete data were required). This approach accounts for minor variations in denominators across tables.

### 2.5. Ethical Considerations

There were no clinical interventions in this study. Responses were anonymous and only used for scientific purposes, with informed consent, in line with research ethics, respecting patient autonomy and confidentiality [31].

The study was conducted in accordance with the Declaration of Helsinki and approved by the Scientific Research Ethics Committee and the Scientific Council of the “Carol Davila” University of Medicine and Pharmacy (UMFCD), Bucharest, Romania.

## 3. Results

The analysis is structured by the following:

- General sample characteristics;
- Scaled item scores (Q20–Q34);
- Significant differences between groups;
- Correlations between items.

Statistical analysis was performed on a total of 397 completed questionnaires, with the number of valid cases varying by item, as detailed in the tables.

### 3.1. General Characteristics of the Sample

Table 1 provides an overview of the socio-demographic distribution of the sample, including variables such as gender, age, place of residence, level of education and marital status. The sample comprises a majority of female respondents and a significant proportion of young people aged 18–24. The majority of participants come from urban areas (92.2%), which is relevant for the present study, given that these areas offer easier access to information and digital technologies. The level of education is also high: more than half of the respondents having received higher education, suggesting a high degree of digital literacy and a greater receptiveness to technological innovations in healthcare. About 47% of participants are married, a social characteristic which may influence attitudes towards modern dental treatments through relational stability and shared decision-making within the family unit.

**Figure 1:**
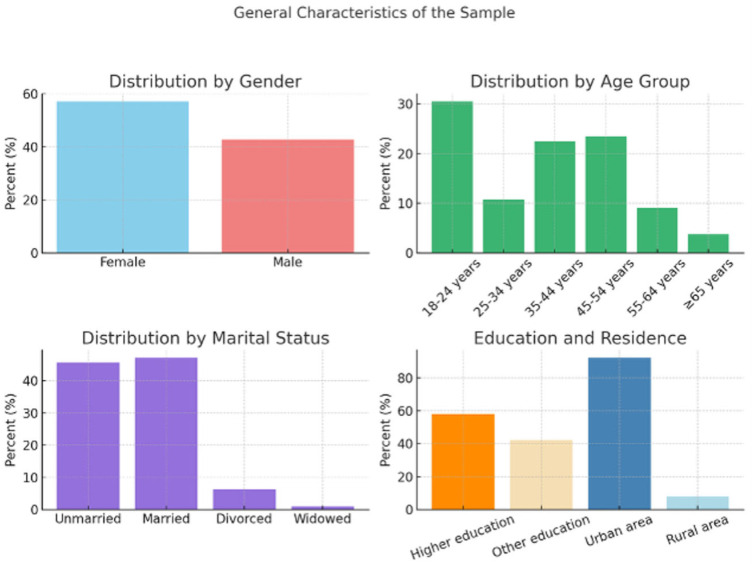
Distribution of respondents by gender, age group, marital status, education and residence.

The visual distribution of these features is shown in Figure 1.

Over 85% of participants have visited the clinic in the last 12 months, and almost a third have undergone treatments such as dental implants, while prosthetics and orthodontic treatments were reported by about half of the respondents.

Although 85.6% of the participants had heard of digitalisation, only 61.2% said they had a clear understanding of it, indicating a significant gap between exposure and actual knowledge. However, 67.3% believe that digital technologies bring tangible benefits, suggesting a positive attitude, despite limited understanding. The most common applications identified by patients include orthodontic treatments (63.7%), surgical planning (56.7%) and digital imaging (54.9%)—areas that coincide with the directions of advanced digitalisation in current practice. The main sources of information are clinical staff (56.9%) and the internet (44.8%), confirming both the essential role of the clinical team in patient education and the importance of an online presence in shaping perceptions of innovation in dentistry.

### 3.2. Attitudes Towards Digital Technologies—Descriptive Scores—Q20–Q34

Table 2 presents the mean scores, standard deviations and medians for the scaled items Q20–Q34. The high values for most of the items indicate an increased level of acceptance and confidence in digital technologies by patients. The most valued aspects are patient comfort, aesthetics of restorations and the perceived safety of treatment. In contrast, the cost-related scores reflect an ambivalent perception, with patients recognising higher costs, but also expressing a relatively good acceptance of these costs if justified by quality.

The highest mean scores were recorded for items Q22 (all dentists should be familiar with the latest technological advances), Q23 (technology improves patient comfort), Q21 (preference for doctors using digital technology), Q26 (perception of a more accurate outcomes/successful treatments) and Q20 (interest in receiving information about technology). Specifically, items Q20–Q22 highlight patients’ proactive attitude towards information and their conscious choice of modern treatments.

These data support the hypothesis that a positive perception of digital technologies extends beyond clinical outcomes to include informational and decision-making dimension, in line with the literature on patient-centred dentistry.

**Figure 2:**
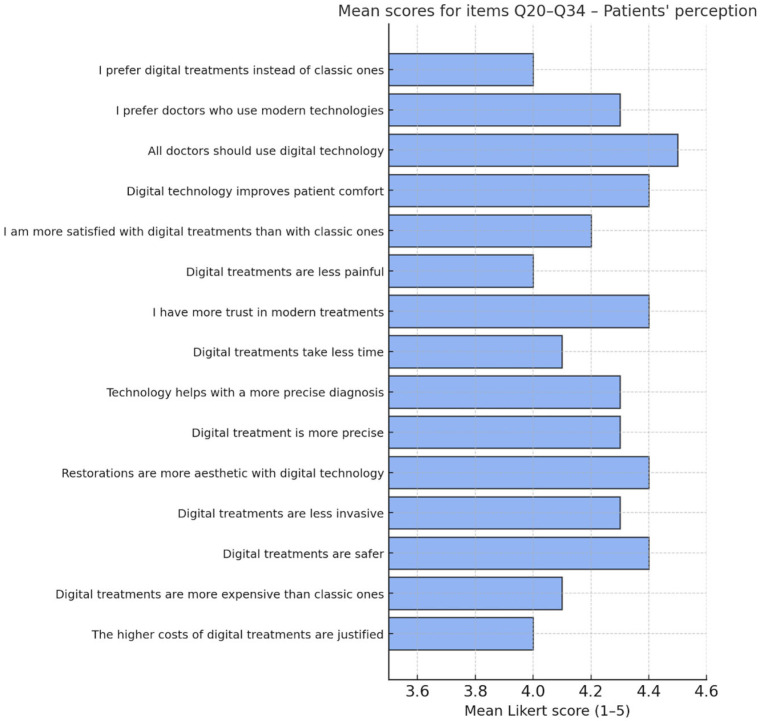
Mean scores obtained for items Q20–Q34, which assess patients’ attitudes and perceptions of digital technologies used in modern dental treatment.

Figure 2 summarises these scores, highlighting the most popular items.

### 3.3. Significant Differences Between Demographic and Clinical Groups

Comparative statistical analyses (Mann–Whitney U and Kruskal–Wallis H) revealed several significant differences in perceptions of digital technologies used in dental treatments between demographic and clinical subgroups. The identified differences are presented below and are organised according to the analysed independent variables.

#### 3.3.1. Differences by Age, Marital Status and Last Visit to the Doctor

Participants aged 45–54 years gave significantly higher scores to item Q29 (accuracy of treatment) than those aged 18–24 (*p* = 0.014—effect size r = 0.226), suggesting that older patients value the accuracy of technology more.

Married individuals gave higher scores to items Q25 and Q29 (*p* = 0.002—effect size r = 0.188 and *p* = 0.013—effect size r = 0.159), indicating a possible relationship between social stability and openness to modern treatments. In contrast, on Q33 (perception of costs), unmarried patients reported higher costs than divorced patients (*p* = 0.016—effect size r = 0.209), which may reflect a different economic perception.

Respondents who had visited the dentist in the past year provided higher scores on Q34 (cost justification) compared to those who had not visited for more than 5 years (*p* = 0.006—effect size r = 0.183), suggesting a correlation between the frequency of visits and the acceptance of costs associated with digital treatments.

#### 3.3.2. Differences by Patient Tenure at the Clinic

Comparative analysis of scores for items Q21 (preference for doctors using modern technologies), Q23 (increased comfort level associated with digital technologies), Q24 (higher satisfaction with conventional treatments) and Q28 (the role of digital technology in facilitating correct diagnosis) revealed statistically significant differences according to how long ago patients first enrolled at the participating clinics. For all four items, scores were significantly higher among patients who had been attending the clinic for less than one year than among those who had been attending for more than five years (Q21: *p* = 0.022—effect size r = 0.185; Q23: *p* = 0.028—effect size r = 0.180; Q24: *p* = 0.020—effect size r = 0.187; Q28: *p* = 0.009—effect size r = 0.203). Additionally, for item Q28, patients with less than one year of clinic attendance had a significantly higher score than those with between 1 and 3 years of attendance (*p* = 0.031—effect size r = 0.185).

These results suggest a greater openness to digital technologies among newcomer patients, possibly reflecting generational trends or recent exposure to modern approaches in clinics. It is also possible that the clinician-patient relationship is more important for the patient than the technical equipment and the infrastructure.

#### 3.3.3. Differences According to the Number of Natural Teeth

The data revealed statistically significant differences for the Q21 score according to the number of natural teeth (*p* = 0.021—effect size ε^2^ = 0.033). Patients with 10–19 natural teeth showed a significantly higher level of agreement than those with all natural teeth (*p* = 0.014—effect size r = 0.229).

This observation may indicate an increased receptiveness to innovation among patients who have experienced tooth loss, possibly due to treatments or in the context of a perceived need for modern solutions.

#### 3.3.4. Detailed Differences by Orthodontic and Prosthetic Treatment History

All analysed items showed statistically significant differences between patients with and without orthodontic (e.g., Q23–Q32, *p* < 0.05) and prosthodontic (Q20–Q32, *p* < 0.05) treatments.

Patients with a history of orthodontic treatment expressed significantly higher levels of agreement:

- Comfort associated with digital technologies (Q23: *p* < 0.001—effect size r = 0.180);
- Positive perception of accuracy, safety and aesthetic outcomes (Q24–Q29);
- Cost justification (Q30–Q32).

Also, patients with a history of prosthetic treatment showed a higher level of acceptance of digital technologies for all items analysed, with significant differences in the perception of functional and aesthetic benefits and added value of modern treatments (e.g., Q24: *p* = 0.010—effect size r = 0.129; Q25: *p* = 0.004—effect size r = 0.144; Q29: *p* = 0.009—effect size r = 0.132).

Respondents who had previously undergone orthodontic, prosthodontic or implant treatment gave significantly higher scores to items Q30 (aesthetics), Q31 (minimally invasive) and Q32 (safety), indicating an increased acceptance of technological innovation among experienced patients.

Patients with previous experience of using digital technologies perceive the clinical benefits of digitalisation more positively, particularly with regard to the aesthetics of restorations, the minimally invasive nature of interventions and the overall safety of treatment.

This trend supports the idea that direct, clinical familiarity with innovation leads to a more favourable attitude towards digitalisation.

### 3.4. Correlations Between Likert-Type Items

The Spearman coefficients for items Q22–Q34 ranged between r = 0.035 and r = 0.695, confirming high internal consistency and conceptual coherence between the analysed dimensions (comfort, aesthetics, safety, costs, satisfaction, etc.). The strongest correlations were observed between items relating to patient comfort (Q23), safety (Q32), cost justification (Q34) and treatment aesthetics (Q30). This validates the existence of a coherent psychometric construct based on positive perceptions of the digitalisation of dental treatment.

For the aggregate agreement indices, associations were as follows: Score-Q33 with other items, ρ = 0.181–0.299 (all *p* < 0.001); Score-Q34 with other items, ρ = 0.343–0.525 (all *p* < 0.001). The largest coefficients were Score-Q33 with Q27 (ρ = 0.299) and Q28 (ρ = 0.289), and Score-Q34 with Q25 (ρ = 0.525), Q23 (ρ = 0.484), Q28 (ρ = 0.493).

Further analysis of correlations by demographic subgroups provides a more nuanced perspective on patient perceptions. Among patients under 35 years of age, strong correlations were noted between items associated with perceived comfort, the aesthetics of restorations, and confidence in technology (e.g., Q23, Q24, Q26, Q30). This association suggests that younger patients prioritise direct benefits over personal experience in digital treatment. At the opposite pole, patients over 45 years of age showed stronger correlations between items reflecting safety, accuracy and cost justification (e.g., Q29, Q32, Q34), indicating an orientation towards clinical effectiveness and the rational validation of therapeutic options.

According to educational level, patients with a secondary education showed strong correlations between diagnostic clarity (Q28) and overall satisfaction (Q24, Q34). In contrast, those with higher education showed higher scores and significant correlations between items reflecting the aesthetic and qualitative evaluation of the final outcome (Q30, Q31), in line with the general trend observed for items Q20–Q21 regarding active involvement and preference for innovation.

These findings support the hypothesis that digitalisation is perceived differently according to demographic profile. Younger patients focus on tangible and experiential benefits, while older patients focus on safety, efficiency, and cost justification.

## 4. Discussion

This study assessed patients’ knowledge of and attitudes towards digital technologies used in modern dental treatment, based on a sample of 397 respondents from three urban dental clinics. Statistical analysis revealed significant differences according to age, marital status, clinical history and length of relationship with the clinic. The majority of the patients (61%) stated that they were aware of digital technologies in dentistry and 67.3% felt that they brought clear benefits to treatment. A similar result was reported by Ayad [7], who revealed a generally positive attitude and high level of awareness among patients regarding the use of advanced technologies, including artificial intelligence, in dental practice. This openness to digital tools has also been observed in other medical fields, with patients generally reporting positive experiences with digital health solutions [6]. However, the level of deep understanding remains partial, with many patients indicating only superficial understanding. A similar phenomenon, but reported among dental practitioners, was described by Hall [32], who highlighted a discrepancy between awareness and practical skills in the use of digital technologies; however, differences in stakeholder group and context mean limit the direct generalisability of any cross-group inferences.

In terms of sources of information, 56.9% of patients stated that they relied on healthcare professionals as their main source, while 44.8% used the internet. This behaviour aligns with the data of Alhomsi [33], which showed that 89.7% of patients consult their doctor at the first sign of a dental problem, compared to only 10.3% who prefer information from social networks.

Differentiated analysis of Likert scores (Q20–Q34) revealed a predominantly favourable attitude towards the use of digital technologies. The highest levels of agreement were recorded for increasing the accuracy of treatment (Q29—87.9% agreement), improving the aesthetics of rehabilitation (Q30—90.7%) and increasing the safety of treatments (Q32—91.4%).

Age was a differentiating factor: respondents in the 18–24 age group showed significantly lower scores for Q29 compared to those in the 45–54 age group (*p* = 0.014). These data partially contradict some trends suggesting that younger patients are more likely to adopt and use digital technologies [34], and may, in our clinical context, reflect familiarity effects, given that technology readiness is associated with greater digital adoption in dentistry [12]. Younger respondents may prioritise how care feels (comfort, aesthetics), whereas older adults tend to weigh safety and accuracy more heavily. Older patients also have more direct experience with complex rehabilitative treatments, which can anchor their judgements of “accuracy” in lived outcomes. Our urban, highly educated sample and unmeasured factors such as objective digital literacy, may also shape this pattern; replication in more diverse settings is advisable.

Education and previous experiences with prosthodontic or orthodontic treatment were found to be relevant predictors of favourable attitude towards digitalisation. Patients with a university education and previous prosthodontic treatment showed higher levels of agreement regarding the efficiency and safety of digital technology use (Q26, Q27, Q28). This trend is supported by Sharka [35], who demonstrated that the perception of a superior clinical experience due to artificial intelligence is a significant predictor of its acceptance, and by the observations of Yi & Choi [36], who showed that previous experience with digital technology positively influences the intention to adopt future innovations. Furthermore, varying levels of familiarisation with digital technologies have been reported even among future dental professionals, emphasising the importance of adequate training from an early stage (as the academic training phase) [37].

Items Q33 and Q34, which focused on the perceived cost of digital treatments, showed a significant correlation between the perceived high cost and its acceptance: 78.6% of respondents believe that digital treatments are more expensive than conventional ones, and 77.6% said that these costs are justified. This correlation supports the hypothesis that patients evaluate price in relation to perceived benefits such as increased accuracy and superior aesthetics. This phenomenon is also reflected in the literature [38,39]. This finding emphasises the importance of effectively communicating the added value of digital technologies to patients, rather than just their price.

There was also a slight variation by marital status, with married and widowed patients showing a higher degree of acceptance of cost justification, which may reflect a more mature perspective on the value of treatments.

Patients recently enrolled (less than one year) in the clinic had significantly higher scores for items expressing confidence in the technology and potential satisfaction (Q21, Q23, Q24, Q28). This result may reflect an ‘initial enthusiasm’ among the newly enrolled patients, supported by the observations of Afrashtehfar [40], who emphasised that patient satisfaction depends heavily on effective management of expectations, clear and encouraging communication, and addressing patient concerns.

### 4.1. Clinical and Policy Implications

Our findings point to clear strategies for bridging the gap between patients’ high exposure to digital technologies and their limited understanding of them. Firstly, it suggests that clinics should ensure that dental staff are trained to clearly explain digital options and include short discussions about how digital tools work during routine visits (e.g., a structured ~90-s chairside script in plain language). As most patients trust their dentist as their main source of information, this step is essential. Secondly, simple videos or infographics may further support comprehension (e.g., a one-page infographic on accuracy, comfort, time, and cost–value; ≤90-s pre-visit videos embedded in appointment reminders). Thirdly, the predominantly urban sample highlights the need to consider how digital workflows can be extended to non-urban settings as well. Finally, patients should always be given clear explanations about real costs and benefits, to address any concerns and mixed feelings about whether digital treatments warrant additional expense (e.g., using a simple cost–value calculator when digital options add measurable benefit; materials should be relevant and kept at a plain-language readability level).

### 4.2. Study Limitations

Design related. As with any cross-sectional, questionnaire-based research design, this study has a number of methodological limitations that need to be taken into account when interpreting the results. The cross-sectional nature of the study only allows associations between variables to be identified, without enabling cause-effect relationships to be demonstrated. Responses were self-reported, which may introduce a social desirability bias.

Sampling-related. The sample was recruited exclusively from three private urban clinics, which generates a possible selection bias: most of the patients interviewed had increased access to modern services, a high level of education and previous familiarity with technological innovations. This limits the extent to which the results can be applied to rural populations, public clinics or disadvantaged socio-economic groups. Although the distribution of the questionnaire was controlled (every third scheduled patient was invited), self-selection could not be entirely ruled out.

Instrument-related. Additionally, the questionnaire did not include reverse-worded questions, which are useful for controlling for confirmation bias. Some questions may be considered suggestive, because they imply a priori benefits of technology (e.g., “Do you think digital treatments are more accurate?”), which could bias responses in a favourable direction and reduce the objectivity of reported perceptions.

Two questionnaires were only partially completed and were included in the analysis only where values were available, resulting in small variations in the number of respondents for some items (e.g., 395 instead of 397). The statistical analysis also identified a sub-group of questionnaires with identical Likert response patterns suggesting possible mechanical completion or lack of engagement, and thus potential response bias. These questionnaires were retained in the overall descriptive analysis but were excluded from the comparative and correlational analyses to maintain the statistical validity of the results.

The questionnaire was internally validated, achieving a Cronbach’s Alpha coefficient of 0.926, indicating excellent internal consistency. However, the lack of external validation using another sample limits the external validity and general applicability of the instrument. Additionally, no items were included that refer to emotional factors such as anxiety about technology, fear of mistakes or trust in medical staff—all of which are relevant variables in patient decision-making. Furthermore, the degree of digital literacy (e.g., frequent use of the internet or medical apps), an aspect significantly associated with patients’ ability to understand and correctly use modern technologies [10], was not objectively measured. This is also confirmed by Valizadeh-Haghi & Rahmatizadeh [11], who showed that digital literacy is directly correlated with patients’ interest in using online health information. Another aspect to be considered is the influence of response style, such as the tendency to choose the extremes of the scale or to agree with most statements, which has been described in the methodological literature, for example, by Weijters [28].

### 4.3. Future Research Directions

The results of the present study open multiple relevant directions for future research in the field of patient perceptions of digital technologies used in dentistry. Firstly, longitudinal studies are needed to assess how attitudes change over time and to correlate initial perceptions with actual post-treatment compliance and satisfaction. Concretely, a prospective longitudinal cohort with baseline attitudes and a 6–12-month follow-up should capture actual uptake of digital procedures, satisfaction, and perceived value, with analyses pre-registered and informed by an a priori power calculation. Such research could contribute to understanding how clinical experience influences patients’ trust in digital technologies. Secondly, expanding the sample to more diverse populations in terms of geography, socio-economic and educational background would allow for a more robust external validation of the instrument and support the development of communication strategies tailored to each patient profile. The inclusion of respondents from rural settings, public clinics or with low levels of digital literacy is essential to obtain a representative picture of technology acceptance in dental practice. A multi-site external validation in public and rural clinics, using stratified sampling (urban/rural; education), should test measurement invariance and differential item functioning to confirm the instrument’s generalisability.

A key area of focus is the development of standardised and externally validated instruments, that assess cognitive, behavioural and emotional dimensions (e.g., anxiety about technology, trust in the medical team, fear of technical errors), as well as providing an objective evaluation of patients’ digital competencies. This can be implemented via a brief task-based digital-literacy screener embedded in the survey. These aspects may have a major influence on the acceptance of innovative treatments and therapeutic decisions.

Also, future studies should distinguish between specific types of digital technologies (intraoral scanning, surgical guidance, CAD/CAM planning, 3D printing, artificial intelligence) and prioritise patient-centred evaluations of specific technologies integral to digital workflows, as perceptions and apprehension may vary significantly depending on the complexity and visibility of each technology in the clinical process. Patient-relevant outcomes should include comfort, anxiety, understanding/comprehension, perceived accuracy, visit burden/time, radiation-risk perception (for CBCT), cost–value trade-off, and equity of access.

The successful implementation of digital dentistry also depends on the skills and attitudes of the medical team, as perceived barriers by professionals may indirectly influence the level of patient acceptance. Finally, integrating emerging technologies, such as dental robotics, could represent the next stage in the expansion of the full digital workflow, requiring further evaluation of clinical effectiveness and acceptance [1,41].

## 5. Conclusions

This survey highlights a generally high level of patient acceptance and interest in digital technologies integrated into modern dental practice, with perceived benefits most frequently linked to increased accuracy, comfort, and aesthetics of prosthodontic restorations [42]. Perceptions vary by age, education, and previous clinical experience, with a more receptive profile among patients with higher education, prior prosthodontic/orthodontic treatment, and a stable relationship with the clinic—underscoring the need for tailored, patient-centred communication that clarifies indications, expectations, and value propositions. Limitations include recruitment from urban clinics only and the use of systematic (non-random) sampling, which may constrain generalisability.

The obtained data support the need for continued integration of digital technologies into therapeutic plans, alongside the development of patient-centred educational strategies. Future studies should longitudinally investigate how repeated exposure to digital treatments influences adherence, satisfaction and long-term clinical outcomes.

## Figures and Tables

**Table 1:** Socio-demographic characteristics of patients.

Parameter	Value (Nr., %)
Survey participant	
Patient	370 (93.2%)
Legal guardian	27 (6.8%)
Gender	
Female	226 (57.2%)
Male	169 (42.8%)
Age	
18–24 years	121 (30.5%)
25–34 years	43 (10.8%)
35–44 years	89 (22.4%)
45–54 years	93 (23.4%)
55–64 years	36 (9.1%)
≥65 years	15 (3.8%)
Civil status	
Unmarried	181 (45.6%)
Married	187 (47.1%)
Divorced	25 (6.3%)
Widowed	4 (1%)
Academic studies	230 (57.9%)
Background	366 (92.2%)

**Table 2:** Distribution of patients by level of agreement for selected items of the questionnaire on digital technology-based dental treatments.

Item (Nr., %)	Highly Disagree	Disagree	Neutral	Agree	Highly Agree
Q20	3 (0.8%)	8 (2%)	85 (21.4%)	172 (43.3%)	129 (32.5%)
Q21	0 (0%)	7 (1.8%)	53 (13.4%)	170 (42.8%)	167 (42.1%)
Q22	1 (0.3%)	2 (0.5%)	24 (6%)	172 (43.3%)	198 (49.9%)
Q23	0 (0%)	1 (0.3%)	35 (8.8%)	168 (42.3%)	193 (48.6%)
Q24	0 (0%)	7 (1.8%)	62 (15.6%)	184 (46.3%)	144 (36.3%)
Q25	1 (0.3%)	9 (2.3%)	119 (30%)	142 (35.8%)	126 (31.7%)
Q26	0 (0%)	3 (0.8%)	41 (10.3%)	196 (49.4%)	157 (39.5%)
Q27	1 (0.3%)	3 (0.8%)	69 (17.4%)	193 (48.6%)	131 (33%)
Q28	1 (0.3%)	3 (0.8%)	40 (10.1%)	202 (51.1%)	149 (37.7%)
Q29	2 (0.5%)	5 (1.3%)	41 (10.3%)	183 (46.1%)	166 (41.8%)
Q30	0 (0%)	2 (0.5%)	35 (8.8%)	177 (44.6%)	183 (46.1%)
Q31	2 (0.5%)	2 (0.5%)	60 (15.1%)	171 (43.1%)	162 (40.8%)
Q32	1 (0.3%)	1 (0.3%)	32 (8.1%)	166 (41.8%)	197 (49.6%)
Q33	2 (0.5%)	5 (1.3%)	78 (19.6%)	193 (48.6%)	119 (30%)
Q34	2 (0.5%)	13 (3.3%)	74 (18.6%)	195 (49.1%)	113 (28.5%)

## Data Availability

The data presented in this study are available on reasonable request from the corresponding author. The data are not publicly available due to patient confidentiality and ethical restrictions.

## Abbreviations

The following abbreviations are used in this manuscript:

UMFCD	“Carol Davila” University of Medicine and Pharmacy
CAD/CAM	Computer-Aided Design/Computer-Aided Manufacturing
Q	Question/Questionnaire Item
CBCT	Cone-beam computed tomography
IOS	Intraoral scanning
SPSS	Statistical Package for the Social Sciences
SD	Standard Deviation
IQR	Interquartile Range
ρ	Spearman’s rank correlation coefficient
